# Butenolide Exerts Antifouling Effects on the Bryozoan *Bugula neritina* through Activating NO/cGMP Signaling Pathway

**DOI:** 10.1093/iob/obag019

**Published:** 2026-04-24

**Authors:** H-L Liu, J-W Qiu, Y H Wong, M-J Yang, J-Q Xie, R-D Xiao, X-L Huang, Y Xu, Z-L Hu, Y Zhang

**Affiliations:** Shenzhen Key Laboratory of Marine Bioresource and Eco-environmental Science, Guangdong Engineering Research Center for Marine Algal Biotechnology, College of Life Sciences and Oceanography, Shenzhen University, Shenzhen 518060, China; Key Laboratory of Optoelectronic Devices and Systems of Ministry of Education and Guangdong Province, College of Physics and Optoelectronic Engineering, Shenzhen University, Shenzhen 518060, China; Shenzhen Key Laboratory of Marine Bioresource and Eco-environmental Science, Guangdong Engineering Research Center for Marine Algal Biotechnology, College of Life Sciences and Oceanography, Shenzhen University, Shenzhen 518060, China; Institute for Advance Study, Shenzhen University, Shenzhen 518060, China; Shenzhen Key Laboratory of Marine Bioresource and Eco-environmental Science, Guangdong Engineering Research Center for Marine Algal Biotechnology, College of Life Sciences and Oceanography, Shenzhen University, Shenzhen 518060, China; Shenzhen Key Laboratory of Marine Bioresource and Eco-environmental Science, Guangdong Engineering Research Center for Marine Algal Biotechnology, College of Life Sciences and Oceanography, Shenzhen University, Shenzhen 518060, China; Shenzhen Key Laboratory of Marine Bioresource and Eco-environmental Science, Guangdong Engineering Research Center for Marine Algal Biotechnology, College of Life Sciences and Oceanography, Shenzhen University, Shenzhen 518060, China; Shenzhen Base of South China Sea Fisheries Research Institute, Chinese Academy of Fishery Sciences, Shenzhen 518116, China; Shenzhen Key Laboratory of Marine Bioresource and Eco-environmental Science, Guangdong Engineering Research Center for Marine Algal Biotechnology, College of Life Sciences and Oceanography, Shenzhen University, Shenzhen 518060, China; Shenzhen Key Laboratory of Marine Bioresource and Eco-environmental Science, Guangdong Engineering Research Center for Marine Algal Biotechnology, College of Life Sciences and Oceanography, Shenzhen University, Shenzhen 518060, China; Shenzhen Key Laboratory of Marine Bioresource and Eco-environmental Science, Guangdong Engineering Research Center for Marine Algal Biotechnology, College of Life Sciences and Oceanography, Shenzhen University, Shenzhen 518060, China

## Abstract

Marine biofouling, a persistent and multifaceted challenge within the maritime realm, generates substantial economic and noneconomic loss and damage to the marine industry every year. Butenolide (BU) is a promising antifouling compound derived from a marine microbe. Although being highly effective against several major biofoulers such as the bryozoan *Bugula neritina*, the molecular mechanisms underlying its inhibition of *B. neritina* larval settlement remain largely unknown. Based on the evidence from comparative transcriptome data, we investigated the expression changes of key genes related to the nitric oxide signaling pathway upon BU treatment via quantitative real-time PCR. Results showed that the expression levels of these genes were significantly up-regulated, suggesting the nitric oxide-cyclic guanosine monophosphate (NO/cGMP) signaling pathway was stimulated in response to BU. In addition, a co-incubation assay using BU and various specific inhibitors targeting the NO pathway showed that these inhibitors could rescue the antifouling effects of BU. These findings further supported the proposition that BU inhibited bryozoan larval settlement by activating the NO/cGMP pathway. Overall, our results provided novel insights into the molecular mechanism of BU against *B. neritina*, which could be crucial for the development of environmentally benign antifouling strategies.

## 中文摘要

海洋生物污损是海洋领域持续存在的复杂挑战, 每年都会给海洋产业带来巨大的经济与非经济损失。丁烯酸内酯 (Butenolide, BU) 是一种来源于海洋微生物的极具潜力的防污化合物。尽管它对包括多室草苔虫 *Bugula neritina* 在内的多种污损生物具有显著抑制作用, 但其抑制多室草苔虫幼虫附着的分子机制仍不清楚。基于比较转录组数据, 我们利用实时荧光定量 PCR 技术分析了BU处理后与一氧化氮信号通路相关的关键基因的表达变化。结果表明, 这些基因的表达水平显著上调, 提示一氧化氮-环磷酸鸟苷 (NO/cGMP) 信号通路在 BU 作用下被激活。此外, 通过 BU 与多种针对NO通路的特异性抑制剂进行共孵育实验发现, 这些抑制剂能够削弱BU的防污效应。上述结果进一步支持了 BU 通过激活 NO/cGMP 信号通路抑制多室草苔虫幼虫附着的观点。总而言之, 我们的研究结果为 BU 抑制多室草苔虫幼虫附着的分子机制提供了新的见解, 这对于开发环境友好型防污策略至关重要。

## Introduction

Marine biofouling is defined as the undesirable colonization of microorganisms, plants, and animals on artificial and natural surfaces submerged in seawater (Yebra et al. 2004). Prominent and detrimental marine fouling organisms include barnacles, oysters, bryozoans, tubeworms, etc. When accumulating on the ship hull, these biofoulers increase hydrodynamic drag and surface corrosion, which in turn elevate fuel consumption and aggravate environmental pollution (Chambers et al. 2006; Brzozowska et al. 2014). Biofouling also facilitates the spread of invasive species and can impair marine infrastructure, posing economic, environmental, and safety risks (Yebra et al. 2004; Kirschner and Brennan 2012; Nurioglu et al. 2015; Xie et al. 2019; Chen et al. 2021). To mitigate these issues, antifouling compounds including tributyl tin (TBT), copper oxide, Irgarol 1051, and Sea-Nine 211 have been widely applied (Alberte et al. 1992; Thomas et al. 2001; Cresswell et al. 2006; Chen and Lam 2017). However, these compounds have the problem of nontarget biological toxicity and environmental persistence (Alzieu 2000; Konstantinou and Albanis 2004; Chen and Lam 2017). Although TBT has been banned by the International Maritime Organization, this chemical persists in sediments and continues to disrupt marine ecosystems. Currently, mainstream antifouling agents, such as copper oxide and cuprous thiocyanate, with supplementation of boosters (Irgarol 1051, Diuron, Sea-Nine 211, etc.), still pose toxicity risks to marine organisms. For example, cuprous thiocyanate has a suppressive effect on the immune system of filter-feeding shellfish such as oysters (Amara et al. 2018), while Irgarol 1051 and Diuron have been directly associated with coral bleaching (Takeuchi et al. 2025). These challenges necessitate the development of nontoxic, eco-friendly antifouling compounds (Xu et al. 2010).

Natural products represent a promising source of eco-friendly antifoulants. Although their mechanisms are still poorly understood, emerging evidence indicates that these anti-fouling agents affect settlement primarily through inhibiting ion channel function, disrupting quorum sensing, blocking neurotransmission, or inhibiting adhesive production or release (Qian et al. 2013). A comprehensive understanding of the mode of action of antifouling compounds is crucial for the identification of key molecular targets and signaling pathways that are critical for the settlement of fouling organisms. This in turn will enable more efficient and precise screening of antifouling compounds (Chen and Qian 2017). According to the Biocidal Products Regulation (BPR) of the European Union, studies clarifying antifouling mechanisms are considered essential for the approval and registration of any new antifouling agent. Therefore, deciphering the mechanisms underlying antifouling processes has become an indispensable step in the development of “nontoxic” antifoulants.

**Fig. 1:**
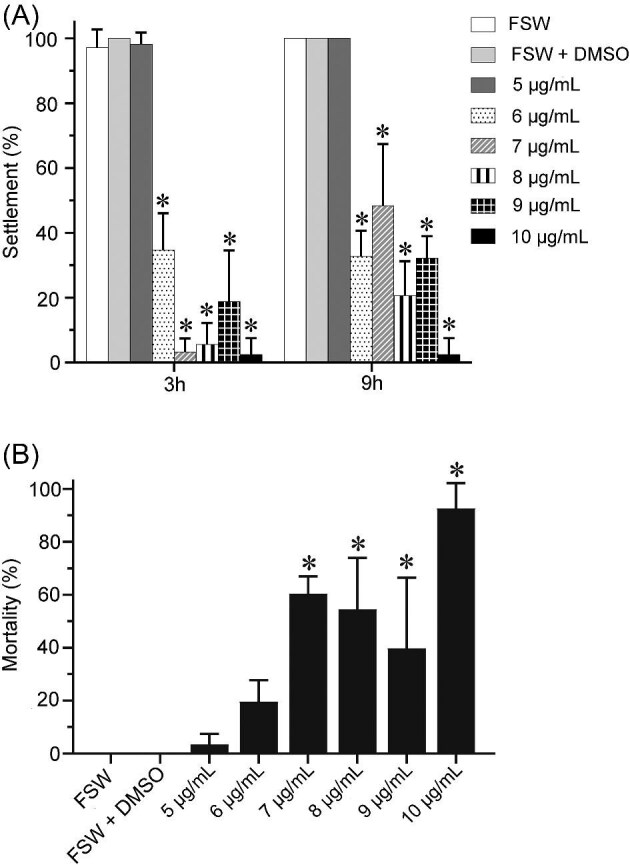
*Bugula neritina* larval settlement bioassay for butenolide treatment. (**A**) Larval settlement rate at 3 and 9 h with different BU concentration treatment. (**B**) Larval mortality at 24 h with different BU concentration treatment. Filtered seawater (FSW) as the control. *Significantly different from the control. * *P* < 0.05.

Butenolide [5-octylfuran-2(5H)-one] (thereafter referred to as BU) is a promising eco-friendly antifouling compound that inhibits larval settlement of major fouling organisms such as barnacles, bryozoans, and tube-building polychaetes (Xu et al. 2010). It exhibits a high therapeutic ratio (LC₅₀/EC₅₀) for larvae of the barnacle *Amphibalanus amphitrite* (>97), the bryozoan *Bugula neritina* (>250), and the polychaete *Hydroides elegans* (>119). Meanwhile, it shows low acute toxicity to nontarget organisms across trophic levels (Zhang et al. 2011b; Chen et al. 2014). However, the molecular mechanisms underlying the antifouling activity of BU against marine fouling organisms are still poorly understood. Previous studies on the barnacle *A. amphitrite* and the bryozoan *B. neritina* (Zhang et al. 2012b) revealed that BU primarily affected proteins involved in energy metabolism. Recently, our transcriptomic analysis of *B. neritina* under BU treatment revealed that, in addition to the up-regulation of genes related to energy metabolism, genes associated with the nitric oxide (NO) signaling pathway were also significantly upregulated (Liang et al. 2022). In this signaling cascade (see Zhang et al. 2012a; Fig. 1), NO is endogenously biosynthesized by a family of NO synthases (NOS), which catalyze the conversion of L-arginine into NO. Subsequently, NO binds to and activates soluble guanylate cyclase (sGC), triggering the conversion of guanosine triphosphate (GTP) into cyclic guanosine monophosphate (cGMP). This second messenger then activates the downstream components and effectors, including cGMP-gated ion channel, cGMP-dependent protein kinase G (PKG), and phosphodiesterase (PDE), and the signal cascade is transduced to cellular responses (Lucas et al. 2000; Friebe et al. 2020). The pivotal role of the NO signaling pathway during larval settlement has been well documented in marine invertebrates across different phyla (Comes et al. 2007; Zhang et al. 2012a; Yang et al. 2018a, 2018b). Thus, we attempted to identify if the NO pathway plays a crucial regulatory role upon BU treatment in the notorious fouling species *B. neritina*.

In the present study, we used *B. neritina*, a marine macrofouling invertebrate with a broad distribution across temperate and tropical waters worldwide (Woollacott and Zimmer 1971), as the experimental model. Specifically, the transcriptional expression patterns of NO signaling pathway-related genes after BU treatment were determined by quantitative real-time PCR. Furthermore, the interaction between the BU and NO signaling was elucidated by co-incubation assays using specific inhibitors of the target molecules and BU.

## Materials and methods

### Larval sampling

Adult *B. neritina* colonies were collected from a fish-farm raft at Dapeng Bay, Shenzhen, Guangdong Province, China (22°33*′*53.2″N, 114°32′09.0″E) and were kept in the laboratory. They were maintained in several plastic tanks (60 × 40 × 30 cm) with continuous aeration supplied by an air pump to ensure adequate oxygenation. Adult colonies were exposed to LED light to induce larval release. For both the BU concentration optimization assay and the specific inhibitor co-treatment assays, newly collected larvae were transferred to 24-well polystyrene plates (Coastar, USA), with each well containing 15–20 larvae in 1 mL of filtered seawater (FSW) or an experimental solution prepared in FSW for a series of pharmacological bioassays. To analyze differential gene expression upon BU exposure, larvae were divided into three experimental groups (FSW control, low-concentration BU treatment, and high-concentration BU treatment). After incubation in the dark for 0.5 h, approximately 800 unsettled larvae from each experimental group were collected, rapidly placed into liquid nitrogen and subsequently stored at –80°C until RNA extraction.

### Optimization of BU concentration for larval settlement bioassays and qPCR analysis

To determine the optimal BU concentrations for both settlement bioassays and subsequent expression analysis by qPCR, preliminary gradient exposure experiments were conducted according to the procedure described in Liang et al. (2022). BU powder (Shanghai Medicilon Inc., Shanghai, China) was dissolved in 100% dimethyl sulfoxide (DMSO) and then diluted with FSW to obtain final concentrations of 5, 6, 7, 8, 9, and 10 μg mL^−1^ BU containing 0.05% DMSO. FSW served as control group. The 24-well plates were kept in dark at room temperature (about 25°C). The number of settled larvae was recorded at 3 and 9 h posttreatment, and larval mortality was recorded at 24 h using a dissecting microscope (Leica EZ4, Heerbrugg, Switzerland). Larvae were deemed dead if they ceased swimming, gradually shrank, and subsequently lysed. The data were analyzed using one-way ANOVA, and multiple comparisons were conducted using Dunnett’s test. Statistical significance was established at *P* < 0.05. All experiments were conducted in quadruplicate.

To obtain early transcriptional responses associated with anti-settlement activity while minimizing the effects of acute toxicity, we selected 6 μg mL^−1^ (low) and 10 μg mL^−1^ (high) BU for qPCR analysis based on previous studies (Liang et al. 2022) and our preliminary experimental results. The low concentration significantly inhibited larval settlement without causing significant mortality. The high concentration induced mortality only after 24 h, while short-term exposure did not cause mortality or impair larval settlement competency (see Zhang et al. 2011a; Liang et al. 2022). In addition, transcriptomic profiles of these two concentrations were highly correlated, suggesting that the core transcriptional responses are primarily associated with anti-settlement activity (Liang et al. 2022).

### Total RNA extraction and reverse transcription for real-time quantitative PCR

Total RNA was extracted using the AxyPrep Total RNA Small Volume Extraction and Preparation Kit (AXYGEN, CA, USA). cDNA was synthesized following the protocol provided in PrimeScript™ RT reagent kit (Perfect Real Time) (TaKaRa, Japan). Based on the comparative transcriptional study of *B. neritina* by Liang et al. (2022), we selected the coding DNA sequences of several differentially expressed genes associated with the NO signaling pathway, namely heat shock protein (HSP90), NOS, and sGC, for real-time quantitative PCR analysis. The primers for these genes are listed in electronic supplementary material. The 18S gene served as the internal reference for normalization, as it exhibits stable expression across experimental conditions and has been widely validated in marine fouling invertebrates, including *A. amphitrite* (Zhang et al. 2012b) and *Hydroides elegans* (Wang and Qian 2010). The relative expression levels of the target genes were calculated using the 2^−ΔΔCt^ method. In the experimental design, the FSW group was used as the control group. The data were analyzed using one-way ANOVA, and multiple comparisons were conducted using Dunnett’s test. Statistical significance was established at *P* < 0.05. Each gene was tested in triplicate.

### Co-treatment of BU and specific inhibitors on *B. neritina*

To confirm the involvement of the NO/cGMP signaling pathway in response to BU treatment, larvae were co-incubated with the lowest concentration of BU, significantly inhibited larval settlement, and different concentrations of specific inhibitors targeting this pathway, including the HSP90 inhibitor geldanamycin (GA), the NOS inhibitors *S*-methylisothiourea sulfate (SMIS) and aminoguanidine hemisulfate salt (AGH), and the sGC inhibitor 1*H*-(1, 2, 4) oxadiazolo [4, 3-a] quinoxalin-1-one (ODQ), respectively. The settlement assay was conducted as described above for the BU concentration optimization assay. Briefly, the number of settled cyprids was counted at 3 and 9 h posttreatment, and larval mortality was recorded at 24 h. Larvae treated with 0.03% DMSO served as the negative controls. All experiments were conducted in quadruplicate. Significant effects on larval settlement and mortality were analyzed by simultaneous multiple comparisons of different means using one-way ANOVA, followed by Dunnett’s test. Statistical significance was established at *P* < 0.05.

## Results

### Bioactivity, toxicity test of BU treatment

As shown in Fig. 1A, when compared with the FSW control, 6 μg mL^−1^ or higher concentrations of BU treatment significantly inhibited larval settlement of *B. neritina* at 3 and 9 h. The larval settlement rate of the 10 μg mL^−1^ treatment reached 2.50 ± 5.00% at both 3 and 9 h. At 24 h posttreatment, the mortality rate in 10 μg mL^−1^ BU treatment group was 92.73 ± 9.51% (Fig. 1B). Since 6 μg mL⁻¹ BU significantly inhibited larval settlement without causing significant mortality, 6 and 10 μg mL⁻¹ were selected as the “low” and “high” BU concentration treatments for qPCR analysis, respectively, while 6 μg mL⁻¹ BU was used in the co-treatment bioassay.

### NO pathway-related genes are highly expressed upon BU treatment

**Fig. 2:**
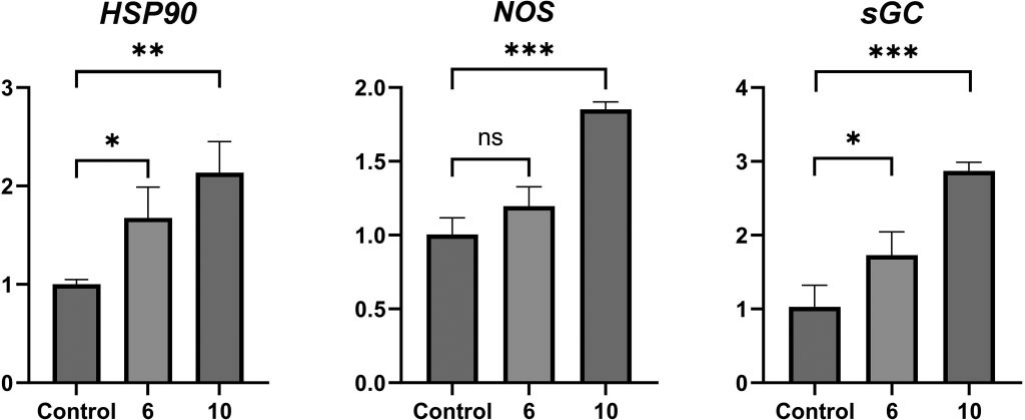
The results of real-time PCR for genes *HSP90, NOS, sGC*, different BU concentration treatment for 0.5 h. Filtered seawater (FSW) as the control. *Significantly different from the control. *P < 0.05, **P < 0.01, ***P < 0.001.

To evaluate the transcriptional changes of genes associated with the NO signaling during BU treatment, three genes related to this pathway, including *HSP90, NOS*, and *sGC*, were selected for quantitative real-time PCR. As shown in Fig. 2, the aforementioned three genes were up-regulated in the 6 and 10 μg mL^−1^ BU treatments, and their expression levels were significantly different from those of the control group when treated with 10 μg mL^−1^ of BU. The expression of *HSP90* was significantly up-regulated at both 6 and 10 μg mL^−1^ of BU treatments, by 1.68-fold and 2.14-fold, respectively. The expression of *NOS* gene was significantly up-regulated by 1.85-fold upon 10 μg mL^−1^ BU-treatment, and *sGC* was up-regulated by 1.73 and 2.87-fold upon 6 and 10 μg mL^−1^ BU-treatment, respectively.

### Reduction of NOS activity rescues the larval settlement inhibition effect of BU

**Fig. 3:**
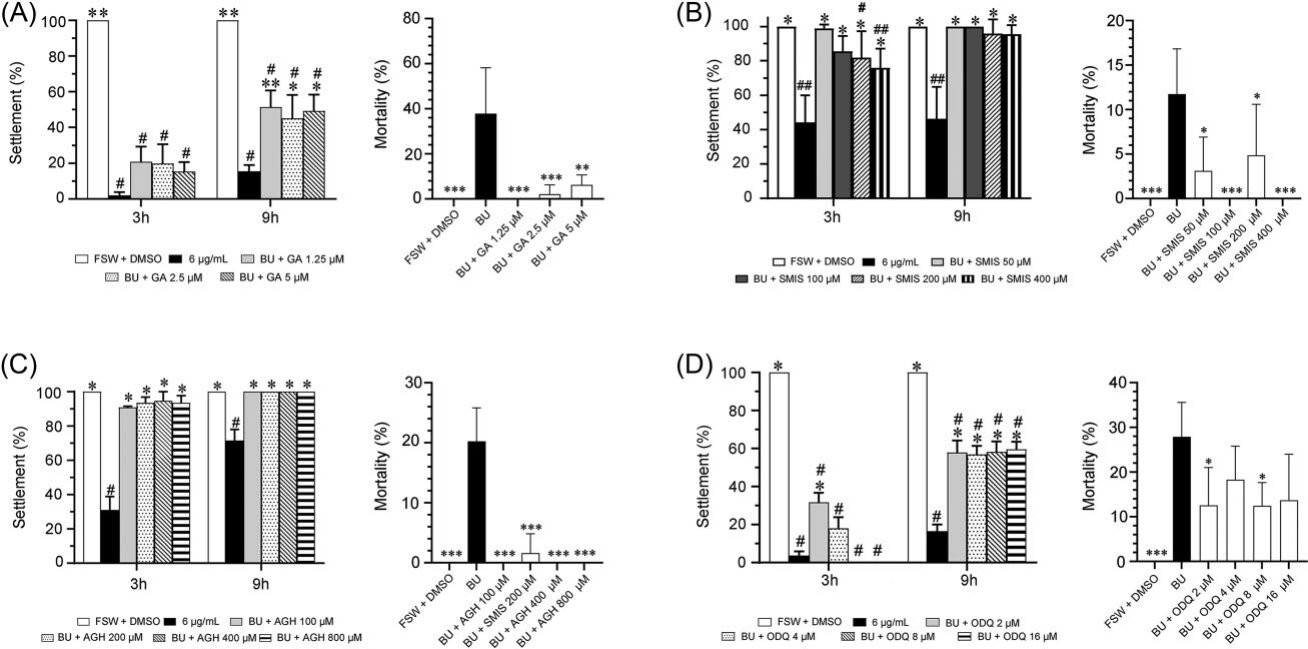
Larval settlement rates at 3 and 9 h, and larval mortality at 24 h in *Bugula neritina* following co-treatment with BU (6 μg mL^−1^) and different concentrations of the HSP90 inhibitors (GA), the NOS inhibitors (SIMS and AGH), and the sGC inhibitor (QDQ). FSW + DMSO (0.03%) as the control for settlement assays, BU (6 μg mL^−1^) as the control for mortality assays. ^#^Significantly different from the FSW + DMSO control. *Significantly different from BU treatment. *^/#^*P* < 0.05, **^/##^*P* < 0.01, ***P < 0.001.

Settlement bioassays were performed to examine whether GA, a potent and specific HSP90 inhibitor (Blagosklonny et al. 2001; Miyata 2005), could attenuate the inhibition of BU during larval settlement. As shown in Fig. 3A, at all tested concentrations (1.25, 2.5, and 5 μM), GA increased the settlement rate to 45.15 ± 26.04 ∼ 51.37 ± 18.68% (*P* < 0.05) in comparison with 15.54 ± 7.05% in the BU treatment group at 9 h posttreatment. After 24-h treatment, the mortality rate in the co-treatment groups (0 ∼ 6.25 ± 4.38%) was significantly lower than that in the 6 μg mL^−1^ BU treatment group (37.81 ± 20.36%) (Fig. 3A).

To examine whether the reduction of NOS activity could rescue the settlement inhibition effect of BU in *B. neritina*, a dilution series of SMIS or AGH was applied together with 6 μg mL^−1^ of BU to the settling larvae, respectively. As shown in Fig. 3B and C, both SMIS and AGH exerted very strong rescue effects at all of the tested concentrations. For SMIS, although the settlement rate of co-incubation groups at 3 h posttreatment (75.78 ± 11.28% ∼ 98.81 ± 2.38%) was slightly lower than their counterparts at 9 h posttreatment (95.58 ± 5.19 ∼ 100%), their settlement rates were significantly higher than that of the BU treatment group (45.27 ± 15.88%). AGH exerted a similar rescue effect, and restored larval settlement to 100% at 9 h posttreatment. As shown in Fig. 3B, the mortality rates in the SMIS + BU co-incubation groups (0 ∼ 4.86 ± 5.73%) were significantly lower than that in the BU treatment group (11.74 ± 5.09%). Notably, the AGH + BU co-incubation groups also exhibited significantly lower mortality (0% ∼ 1.61 ± 3.23%) compared to the BU treatment group (20.24 ± 5.58%) (Fig. 3C).

### Inhibition of soluble guanylyl cyclase activity rescues the larval settlement inhibition effect of BU

To evaluate whether the inhibition of larval settlement by BU is via NO/cGMP signaling, *B. neritina* larvae were co-incubated using BU and sGC inhibitor ODQ. As shown in Fig. 3D, in ODQ (2 μM) + BU co-incubation group, larval settlement was significantly increased (*P* < 0.05) at 3 h posttreatment compared with the group treated with BU alone. Lower settlement rates were observed at higher concentrations of ODQ (4, 8, and 16 μM) in the co-incubation treatments. However, at 9 h posttreatment, settlement rates for all co-incubation treatments were significantly higher than that of the BU treatment group, reaching 56.89 ± 9.12 to 59.54 ± 8.17%. At 24 h posttreatment, the mortality rates in the ODQ (2 μM) + BU co-incubation group (12.56 ± 8.45%) and ODQ (8 μM) + BU co-incubation group (12.45% ± 5.19%) were significantly lower than that in the BU treatment group (27.88% ± 7.68%) (Fig. 3D).

## Discussion

Previous proteomic studies have demonstrated that the antifouling mechanism of BU is associated with energy metabolism. For instance, BU binds to acyl-CoA: cholesterol acyltransferase 1 (ACAT1) in the barnacle *A. amphitrite* and to very long-chain acyl-CoA dehydrogenase-like (ACADVL) in the bryozoan *B. neritina* (Zhang et al. 2012b). Recently, our transcriptome analysis of *B. neritina* under BU treatment showed that, in addition to the up-regulation of genes encoding ACADVL, genes related to the NO signaling pathway (e.g., *NOS* and *sGC*) were also significantly up-regulated (Liang et al. 2022). However, the functional implications of this observation remained unexplored. In this study, to validate the quantitative results reported in the transcriptome analysis, three NO signaling pathway-related genes, namely *HSP90, NOS*, and *sGC*, were chosen for real-time quantitative PCR analysis. In addition, settlement bioassays were conducted to determine whether the pathway-specific inhibitors (HSP90 inhibitor GA, NOS inhibitor SMIS and AGH, and GC inhibitor ODQ) could rescue the inhibitory effect of BU against larval settlement.

Our results revealed that BU treatment upregulated the transcriptional levels of *HSP90, NOS*, and *sGC*, which are all related to the NO signaling pathway and its associated components. HSP90 is a molecular chaperone that binds to NOS and thus maintains and/or enhances its activity (Billecke et al. 2002). The interplay between HSP90 and NOS has been shown to regulate larval settlement or metamorphosis in a variety of marine invertebrates, such as sea urchins (Bishop and Brandhorst 2001), ascidians (Bishop et al. 2002), and bryozoans (Yang et al. 2018a). Notably, a previous study demonstrated that BU treatment also enhanced HSP90 protein expression in *A. amphitrite* (Zhang et al. 2010), aligning with our results of increased transcription level of *HSP90* upon BU treatment in *B. neritina*. These findings suggest that HSP90 is involved in modulating NO pathway in response to BU.

In *A. amphitrite*, it has been demonstrated that NO regulates larval settlement via mediating downstream cGMP signaling (Zhang et al. 2012a). In the present study, *HSP90* (molecular chaperone of NOS), *NOS* (the NO producer), and *sGC* (the downstream receptor of NO) were all up-regulated in response to BU treatment, which is in agreement with our previous transcriptome analysis, and further supports the activation of the NO/cGMP pathway at the transcriptional level. Up-regulation of components in this pathway was also observed for another antifouling compound, cochliomycin A, which inhibits larval settlement of *A. amphitrite* by activating the NO/cGMP pathway (Wang et al. 2016). In addition, the NO/cGMP pathway appears to be a conserved regulatory mechanism across different phyla, as it has been reported to inhibit metamorphosis or settlement in diverse taxa, including the gastropods *Crepidula fornicate* (Pechenik et al. 2007) and *Phestilla sibogae* (Bishop et al. 2008), the sea urchin *Lytechinus pictus* (Bishop and Brandhorst 2001), the ascidians *Boltenia villosa* and *Cnemidocarpa finmarkiensis* (Bishop et al. 2002), and *Ciona intestinalis* (Comes et al. 2007), as well as the barnacle *A. amphitrite* (Zhang et al. 2012a) and the bryozoan *B. neritina* (Yang et al. 2018a, 2018b). Taken together, our findings suggest that the NO/cGMP pathway is a potential molecular target mediating BU’s antifouling activity, highlighting the prospect of targeting this evolutionarily conserved pathway for the development of novel and effective antifouling agents.

The involvement of the NO/cGMP pathway upon BU treatment is further supported by the following evidence from co-treatment experiments: (1) The partial restoration of larval attachment in the co-treatment group of HSP90 inhibitor GA and BU is likely attributed to GA-mediated reduction in NOS stability and activity, thereby decreasing NO production. (2) Co-treatment of NOS-specific inhibitors SMIS/AGH and BU, which presumably suppresses NOS activity potentially induced by BU, reduced endogenous NO levels, thereby attenuated the larval settlement-inhibitive effect of BU. (3) Combined application of the sGC inhibitor ODQ and BU resulted in significantly higher settlement rates compared to BU treatment alone, suggesting the suppression of cGMP production through inhibition of sGC activity can also alleviate the inhibitive effect of BU.

The co-treatment experiments also showed that when settlement capability was restored, larval survival of all co-treatments significantly increased compared to the BU solo treatment group. These results collectively indicate that the survival of *B. neritina* larvae can be rescued by modulating NO signaling-related components, implying that the lethal effects should not be a result of direct BU toxicity, but rather stem from indirect physiological response, particularly delayed settlement/metamorphosis. Hunter et al. (1998) showed that settlement and metamorphosis success of bryozoan *B. neritina* (L.) were significantly reduced after 24 h of swimming. Similarly, in another bryozoan *Bugula flabellate*, the metamorphosis success was significantly decreased from 100% to around 50% and 20% when larvae experienced prolonged swimming for 6 and 12 h, respectively (Cancino and Gallardo 2004). By restoring settlement via inhibition of pathway components, larvae complete metamorphosis and evade mortality, explaining the reduced death rates in co-treatment groups. This resolves the initial ambiguity about BU’s toxicity and emphasizes the importance of physiological context in interpreting antifouling compound effects.

## Conclusion

Our study demonstrates that BU inhibits *B. neritina* larval settlement by activating the NO/cGMP signaling pathway, supported by the transcriptional up-regulation of HSP90, NOS, and sGC, as well as functional rescue by pathway-specific inhibitors. However, given that our qPCR analysis was limited to hypothesis-driven candidate genes associated with the NO/cGMP signaling pathway, rather than a global set of differentially expressed genes, and considering that pharmacological agents may exert off-target effects, we cannot rule out contributions from additional signaling pathways, either independently or in concert with NO/cGMP signaling.

Given the well-documented role of NO signaling pathway in regulating the larval settlement across different phyla of marine invertebrates, BU may possess broad-spectrum antifouling potential. However, this conclusion can only be drawn when parallel analyses are conducted using sufficient number of representative species from different phyla, particularly those that are ecologically prominent in marine biofouling communities, such as barnacles (e.g., *A. amphitrite*) and mussels (e.g., *Mytilus edulis*). To fully characterize the molecular mechanism of BU, more detailed functional analyses of the specific molecular targets identified in this work should be conducted. Additionally, the possible crosstalk between NO signaling and other signaling pathways in response to BU treatment also warrants further exploration.

## Supplementary Material

obag019_Supplemental_File

## Data Availability

All relevant data and details of resources can be found within the article and its supplementary information.
