# Supplementary material for: Butenolide Exerts Antifouling Effects on the Bryozoan Bugula neritina through Activating NO/cGMP Signaling Pathway
Source: Integr Org Biol. 2026 Apr 24;8(1):obag019. doi: 10.1093/iob/obag019 (PMC13191283; doi:10.1093/iob/obag019)
Supplement: obag019_Supplemental_File [file obag019_supplemental_file.docx]

Table S1. Primers used for real-time PCR in this study

| Gene name | Primer direction | Primer sequence (5′→3′) |
| --- | --- | --- |
| *NOS* | Forward | TCTGCATCACAAGGGACACC |
|  | Reverse | ATTCACACCAATGCCTGGCT |
| *sGC* | Forward | GCATGGTGGAGTTGTGGAGA |
|  | Reverse | TCTTGCCAGGTACACCGAAC |
| *HSP90* | Forward | CCTGACACTGATCGACACCG |
|  | Reverse | ACCCACACCAAACTGTCCAA |
| *18S* | Forward | CCGGCGACGCCTTCACTGAG |
|  | Reverse | CGCGCCTGCTGCAAACCTTG |
